# Building trust in rural communities: recruitment and retention strategies in developmental science

**DOI:** 10.3389/fpubh.2025.1586988

**Published:** 2025-05-07

**Authors:** Ava Reck, Lauren Holley, Kyle Bower, Sarah Whitaker, Caroline Hall, Courtney Brown, Alison Berg, Caroline Alvarado, Diane Bales, Katharine Suma, Kimberly Fowler, Charles Geier, Assaf Oshri

**Affiliations:** ^1^Department of Psychology, University of Oregon, Eugene, OR, United States; ^2^Department of Human Development and Family Science, University of Georgia, Athens, GA, United States; ^3^Extension and Outreach, University of Georgia, Athens, GA, United States; ^4^Department of Nutritional Science, University of Georgia, Athens, GA, United States; ^5^Department of Psychology, New York University, New York, NY, United States; ^6^Office of Research, University of Georgia, Athens, GA, United States

**Keywords:** building trust, rural communities, recruitment strategies, retention strategies, developmental science, community engagement, rural engagement, recruitment and retention

## Abstract

**Background:**

Studying human development often requires intimate interpersonal queries and interactions with children and families. Such research necessitates moving beyond traditional lab settings to engage participants within their communities for an extended period of time. Building trust is essential for conducting ecologically valid, longitudinal research, particularly when working with diverse and historically underserved populations. Developing effective, practical strategies to foster trust and rapport enhances recruitment, retention, and the overall quality of developmental research. This manuscript examines the recruitment and retention strategies aimed to facilitate trust and engagement in a longitudinal study involving rural participants in Northeast Georgia.

**Methodology:**

The continuum of community engagement model suggests that research recruitment efforts should involve more than direct participant outreach, toward a multifaceted, community-driven approach. The current study employs a mixed-methods framework to evaluate these strategies in fostering trust and participant engagement. Qualitative data was collected from study reflection notes, interviews with extension agents, and participant surveys, alongside quantitative measures tracking enrollment, contacts, referrals, and participant trust.

**Results:**

Our findings suggest that trust-building efforts, including community engagement teams, reciprocal community relationships, family and family-centered environments, and tailored communication strategies, enhanced participant engagement. Referrals emerged as the most effective method for recruitment. Integrating community-driven recruitment methods led to increased referrals and fostered long-term relationships and trust with community partners, but this success was only achieved after establishing connections and building trust within the community.

**Conclusion:**

Addressing historical mistrust and promoting research inclusivity requires iterative, community-centered approaches. Our study highlights the importance of transparent communication, adaptive recruitment strategies, and sustained community engagement. Findings provide a framework for future research aiming to enhance trust, recruitment, and retention in complex biomedical and behavioral developmental science, ensuring more representative and impactful studies.

## 1 Introduction

Developmental science is a rapidly expanding interdisciplinary field that examines how biological, psychological, and social factors interact to shape development across the lifespan (1). Addressing transdisciplinary questions about human development often requires complex longitudinal research designs and activities that incorporate interpersonal interactions with families and children over time (2). Participant recruitment (initial study enrollment) and retention (sustained participation) are critical components of human research efforts, particularly for longitudinal developmental studies. In order to obtain reliable and valid rapport, as well as successful recruitment and retention, this research relies heavily on building trust with the participant population (3, 4). However, public trust in science continues to erode (5, 6). As a result, research teams face the increasing challenge of building trust within communities that may have broad socio-historical perceptions of science and research. This challenge necessitates a deeper understanding of the barriers to trust and engagement, as well as the factors that can foster meaningful collaboration between researchers and communities. Discussing and evaluating strategies for recruiting and retaining participants in developmental science can advance efforts to improve the quality (e.g., ecologically valid methodologies; community involvement) and quantity (e.g., increasing participant enrollment and sustaining engagement over time) of scientific research.

### 1.1 Trust and engagement with research

Trust is a critical social process that facilitates cooperation and underlies all human interactions (7). However, trust in scientific institutions in the United States has significantly declined in the 21st century. A 2022 survey revealed a 10% decline in the number of people who said they had confidence in scientists to act in the public’s best interest, dropping from 39% in 2020 to just 29% in 2022 (5). This decline is likely fueled by increased political polarization, the rapid dissemination of misinformation, and other cultural forces that have reshaped public perceptions of science (5, 8).

Mistrust in science may also stem from adverse and unethical research practice and the long-lasting impression these malpractices left in communities. For example, the infamous Tuskegee Syphilis Study, Statesville Penitentiary Malaria Study, Plutonium Trials, and James Marion Sims research on female reproduction are just a few examples of historically relevant studies in which United States public health researchers deliberately deceived participants from vulnerable populations and caused deaths, spread infections, and enacted physical pain [see (9) for a review]. These scientific atrocities still influence mistrust in science today (10).

Although significant progress has been made in the ethical and responsible conduct of research, individuals are still vulnerable to negative experiences with researchers or research institutions in their communities. People may perceive research as predatory, especially when conducted among minoritized communities (11). This may be due to the practice of researchers collecting data without making meaningful investments in the community. A study found that, among 109 community leaders involved in research, 75% viewed researchers as unprepared to engage communities, and 87% reported insufficient resources were available to support community involvement (12). These challenges highlight the urgent need for transparency, accountability, and efforts to rebuild trust between scientific institutions and the communities they serve, particularly if these communities are underrepresented in research.

#### 1.1.1 Trust of research and researchers in rural communities

Over several decades, rural residents have consistently demonstrated low confidence in scientists (13). A recent study found that rural Americans report significantly more negative or “colder” feelings toward scientists than those from urban areas, even while controlling for possible confounds such as political views and media habits (14). Perceptions of research in small communities are also often shaped by the broader relationship between the university and surrounding areas (i.e., town-gown relationships), where tensions surrounding institutions of higher education, such as concerns about student behavior, housing pressures, and competition for shared resources, can strain community relations and foster skepticism toward institutional research efforts (15).

Rural populations may view scientific institutions as distant and disconnected from rural life, showing a “lack of respect” for and dismissing “local knowledge” [(16), p. 126–127]. These negative views toward scientists may be associated with reduced trust in them (17), thus requiring necessary effort and investment in rural communities to understand and develop meaningful, trustful relationships within them. Building relationships and gaining community trust in some rural communities can take years or even decades (14).

#### 1.1.2 The role of trust in research participation

Trust plays a critical role in shaping individuals’ decisions to participate in research. When communities have confidence in researchers and institutions, they are more likely to engage with research and view participation as safe, ethical, and beneficial (18). This may be particularly true in marginalized communities, where past exploitation and ongoing disparities reinforce concerns about being mistreated or misrepresented in research (11, 14). Rurality is recognized as a characteristic of special populations by the Clinical and Translational Science Awards, referring to groups that face unique health challenges (19, 20). People living in rural areas often experience higher rates of isolation and limited access to resources, contributing to increased social vulnerability (20). When a population has sociohistorical experiences that have resulted in a mistrust of research institutions and researchers, more emphasis must be placed on building a genuine and mutual relationship between the individual researchers and the study participants and their community (18).

While there is a wealth of guidance on building trust [e.g., (21, 22)], translating these recommendations into actionable strategies remains challenging. Developing practical, effective approaches to engage all populations is essential for enhancing recruitment, retention, and the overall quality of developmental science. This manuscript aims to describe and evaluate the recruitment and retention strategies aimed at building trust and rapport in a research study recruiting rural participants throughout Northeast Georgia.

## 2 Materials and methods

### 2.1 Background of the parent research project

The current manuscript is based on recruitment and enrollment data from the first year of a longitudinal National Institutes of Health (NIH)-funded study, Building Resilience and Nurturing Children’s Health (BRANCH; NIDA-R01 DA055630). BRANCH, referred to as the parent study in this manuscript, is a prospective, multi-modal, and multi-level research project that engages with rural communities to understand the developmental ecology in which adolescent risk behaviors and resilience emerge during development, spanning childhood to adolescence. BRANCH operates within 12 rural counties in Northeast Georgia. These counties have an average food insecurity rate above the state average (23), are classified as having a shortage of mental health care professionals (24) and report median household incomes below the state median (25). BRANCH is based out of the Georgia Center for Developmental Science, an interdisciplinary center dedicated to understanding the factors shaping youth and family experiences related to risk and resilience. The parent and current studies were approved by the Institutional Review Board (IRB) at The University of Georgia.

#### 2.1.1 Research protocols of the parent research project

BRANCH involves children and their caregivers in the research. Caregivers are defined as the legal guardian of the child. BRANCH includes four waves of data collection, approximately 18 months apart. BRANCH is currently in the first wave of data collection, which started in November 2023 and concludes in the summer of 2025. Data collection first consists of a home visit where the caregiver and child complete surveys, play interactive games, are video recorded, and provide biometric data (e.g., electrocardiogram and eye-tracking). Families then visit the university for a 37-min magnetic resonance imaging (MRI) scan. Before the scan, the child and caregiver provide a hair sample, biometric data, and complete surveys. The visit also includes MRI practice using a mock scanner and a virtual reality headset.

#### 2.1.2 Participant eligibility in the parent research project

Children and their caregivers were eligible for participation in wave one of the BRANCH study if the child was between the ages of six and ten, resided within a rural catchment area within 60 miles of the university campus, and was low-income at the time of recruitment. Rurality was based on the United States Department of Agriculture (USDA) rural classification system (26). Low-income status was based on family income at or below 200% of the federal poverty level (27). Exclusion criteria for child participants included developmental delays (e.g., autism), use of psychotropic medications (self-report), and standard MRI contraindications (e.g., metal implants, claustrophobia). Exclusion criteria for caregivers included a lack of literacy skills sufficient to follow written instructions. For completion of wave one of the study, caregivers received compensation of up to $155 per child, and child participants were compensated with $10 and a toy valued at $10 for participating in each visit. Caregivers were also offered $50 for every successful referral of another family to participate in the study (i.e., the referred participant enrolls in the study).

Notably, the research team adjusted eligibility in March of 2024. This was an effort to maintain the focus on rural populations while expanding the pool of eligible participants to boost enrollment. New eligibility expanded the inclusion criteria to any income level. The research team still focused on the recruitment of participants who were predominately low-income. Participants were recruited through flyers, social media, community event outreach, clipboarding (research staff signing up families at various events), and referrals. Additional recruitment details are provided in the sections below.

### 2.2 Community, family, and participant engagement

During wave one of data collection, the BRANCH team aimed to actively engage communities, families, and participants in the research. The team employed a continuum model of community engagement (28) and strives to advance further along the continuum as the project progresses and capacity grows. The following sections describe the research team, and the protocols designed to promote trust and engagement.

#### 2.2.1 The research team

Building community trust requires a strong team of researchers and staff committed to increasing community engagement. To conduct a study that prioritizes collaboration with the community while maintaining rigorous scientific standards, the BRANCH team established a multi-faceted research team. This team included principal investigators and a project coordinator to oversee research protocols, the Family and Community Engagement (FACE) team to foster community connections, and a Certified Child Life Specialist to ensure families have positive experiences after enrollment. The following sections provide more detail on the FACE team and Certified Child Life Specialist.

##### 2.2.1.1 The family and community engagement (FACE) team

To initiate and build community engagement, the BRANCH FACE team was established prior to data collection. The FACE team consisted of extension agents, a community engagement specialist, community liaisons and a recruitment and communication coordinator. The FACE team worked directly with the project coordinator, who was responsible for reporting progress to the principal investigators.

Extension agents operate out of the Cooperative Extension Service, through the United States Land-Grant University System and in collaboration with federal, state, and local governments, as well as the USDA National Institute of Food and Agriculture [NIFA; see (29) for more information]. Extension agents are county-based personnel employed by university systems to serve local communities and operate as liaisons between researchers and the community. They leverage their local knowledge and relationships to facilitate engagement, recruitment, and the implementation of research initiatives. To engage with the community in Georgia, extension agents deliver educational programs for youth and adults through three main program areas: Family and Consumer Sciences (FACS), 4-H Youth Development, and Agriculture and Natural Resources. In the BRANCH study, extension agents leveraged long-standing relationships within the communities to help identify mutually beneficial and equitable opportunities for collaboration between the communities and the research team.

In the first wave of data collection, the community engagement specialist’s role was to build and maintain relationships with community organizations and liaisons. Community liaisons are trusted individuals, often identified through initial community connections, who help bridge the gap between the study team and the community. In the BRANCH study, liaisons, such as non-profit leaders, librarians, and family-based organization leaders, were compensated $50 for each successful referral into the study. The community engagement specialist was responsible for identifying these community organizations and liaisons, initiating contact, and determining how the study can best engage with and give back to these communities.

The recruitment and communication coordinator was responsible for recruiting and enrolling families and maintaining long-term connections with participants and their communities. They focused on educating families about the BRANCH study, sharing recruitment information, and connecting with participants. Together, the community engagement specialist and recruitment and communication coordinator team members collaborated with local leaders, attended community events, and established consistent lines of communication to enhance engagement.

##### 2.2.1.2 Certified child life specialist

**Figure 1:**
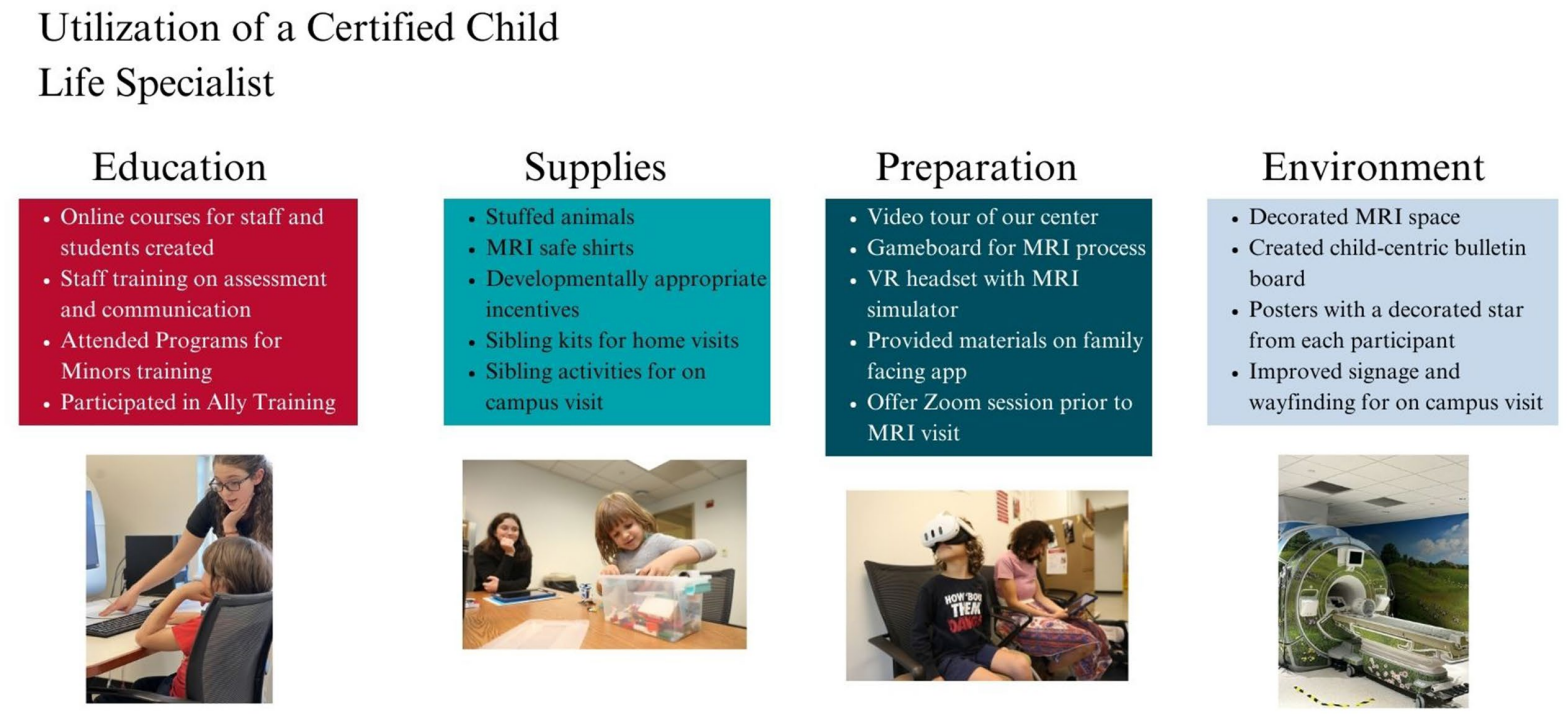
Summary of family-centered interventions being implemented by the Certified Child Life Specialist. Consent was obtained for the use of all participant images depicted in this figure.

Certified Child Life Specialists are formally trained to help reduce anxiety in children and families by co-developing coping strategies and using developmentally appropriate education, preparation, and play (30). Due to potentially new and challenging experiences for young children, the BRANCH research team relied on a Certified Child Life Specialist to establish rapport with families before, during, and after their participation and ensure comfortability with all stages of the study. In BRANCH, the Certified Child Life Specialist took an advisory role in adapting all protocols and resources to meet the child’s needs (see Figure 1 for an overview).

The Certified Child Life Specialist focused on training to prepare all BRANCH staff and students to effectively communicate with children and families (see Figure 1, “Education”). They also introduced innovative technology, such as the personalized SupportSpot app by Child Life on Call,^1^ which provided study details, answers to frequently asked questions, staff introductions, video tours, and MRI sound examples. The app acted as an additional connection point to enhance communication and familiarity with the study and provided families with quick and easy access to resources.

The Certified Child Life Specialist also worked toward enhancing participant comfort by procuring supplies and implementing activities like one-on-one Zoom preparation sessions, interactive games, mock MRI protocols, and a virtual reality MRI simulator (see Figure 1, “Supplies” and “Preparation”). Additionally, the Certified Child Life Specialist improved the physical space with child-friendly elements such as a nature-scene mural, carpets, and stuffed animals (see Figure 1, “Environment”), making the setting less clinical. These strategies were aimed to help reduce anxiety, especially for children unfamiliar with research environments. Overall, the Certified Child Life Specialist used the innovative application of child life skills to create a developmentally appropriate and engaging research experience for BRANCH participants.

##### 2.2.1.3 Research team structure and synergy

**Figure 2:**
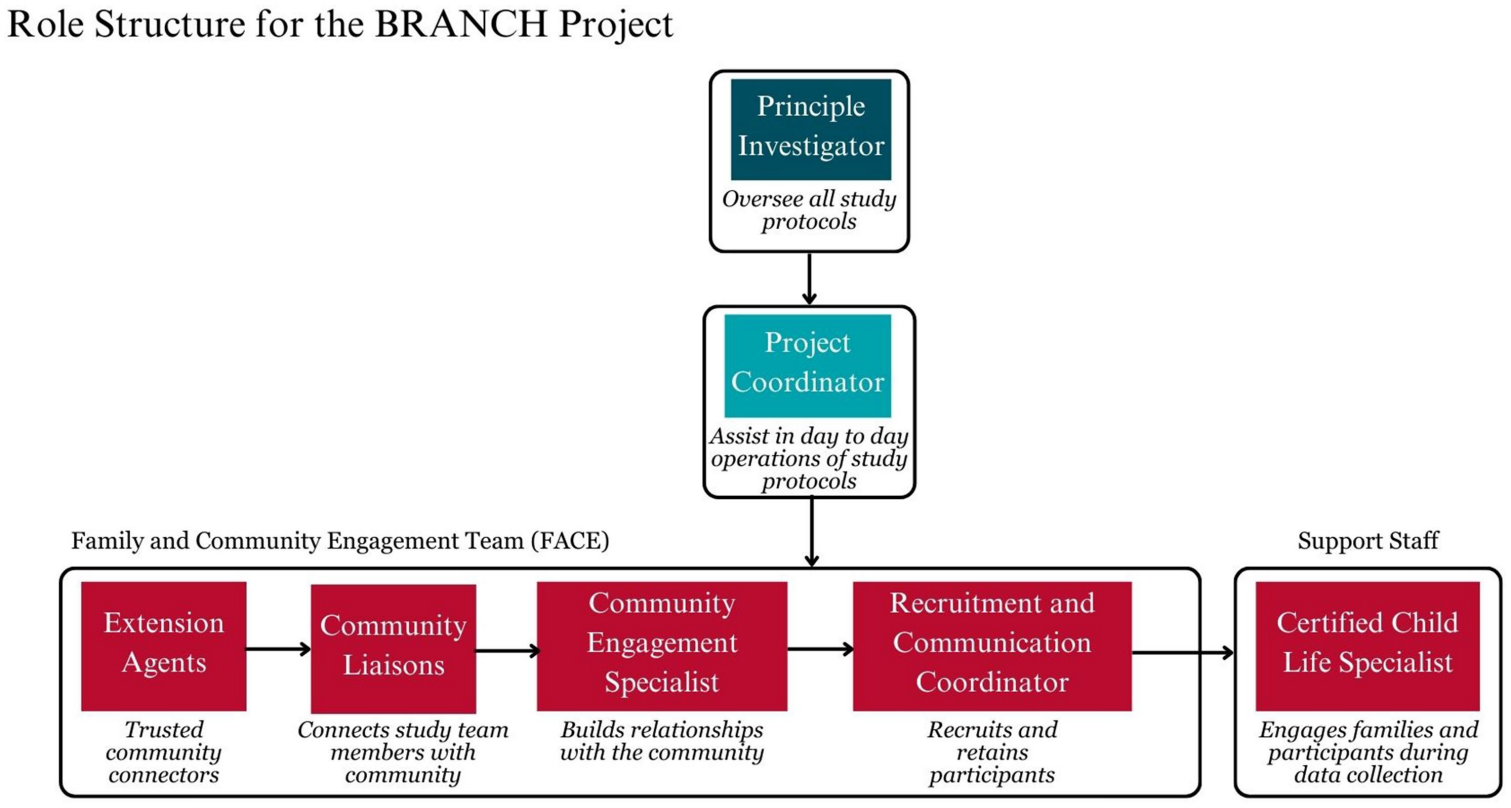
The role structure of the research team in the branch study.

The role structure and synergy of the team can be seen in Figure 2. The principal investigator oversaw all protocols, while the project coordinator managed and supported day-to-day operations. The FACE team engaged the community, families, and participants in the research. This process began with extension agents who, as established members of the community, bridged the initial contact between community liaisons and the community engagement specialist. After the community engagement specialist built these relationships, the recruitment and communication coordinator facilitated family enrollment in the study. Once families were enrolled, the Certified Child Life Specialist provided support to ensure their comfort during data collection, with the goal of increasing referrals and retention. Although this manuscript describes protocols during the first wave of data collection, the FACE team and Certified Child Life Specialist will remain active for the remainder of the study.

#### 2.2.2 Community, family, and participant engagement protocols

**Figure 3:**
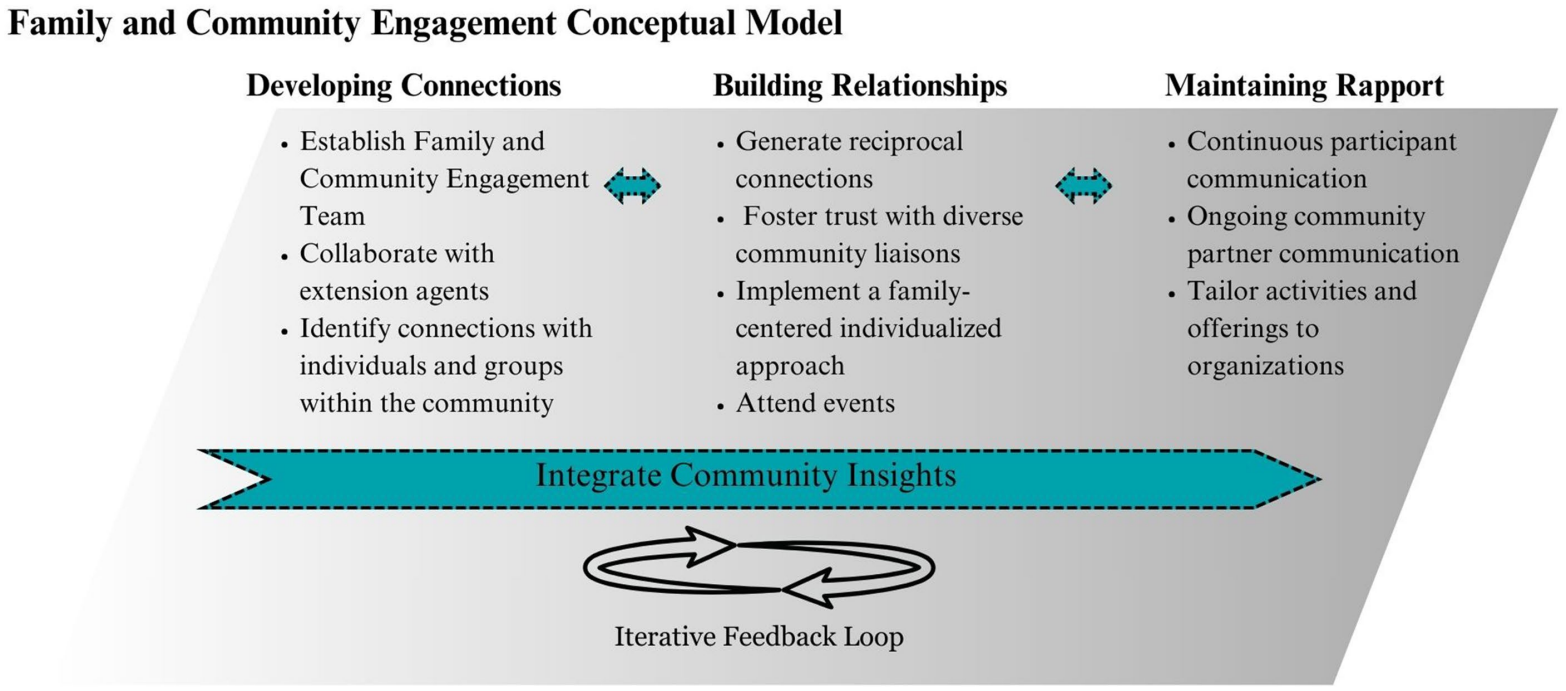
Community, family, and participant engagement process.

Figure 3 visually illustrates the process of community engagement protocols developed by the research team. The protocols are organized into three main categories based on the goals of engaging the community, participants, and families: (1) developing connections, (2) building relationships, and (3) maintaining rapport. These categories are described in more detail below. Importantly, while specific activities align with various categories, the process is highly connected and iterative, represented by dotted arrows between categories. Many protocols operate in a continuous cycle where input is used after each phase to adjust and improve other protocols, represented in Figure 3 as a iterative feedback loop. Community insights are represented by a permeable arrow, symbolizing the ongoing dialogue and adaptation based on insights from the community that occur in every category.

Although the process is iterative, the protocols are presented chronologically to highlight the cumulative factors that contribute to success in developing connections, building relationships, and maintaining rapport. Each category is shaped by time, internal factors (e.g., staffing and project goals), and external factors (e.g., collaborators, communities, and families).

##### 2.2.2.1 Developing connections

During the first 6 months of the project, the principal investigators identified local personnel to join the FACE team and bolster the recruitment network. BRANCH then defined these communication channels to encourage strong role development among FACE staff to cultivate a deep understanding of community needs and values. This included defining the partnership with extension agents formally through a letter of support built by both parties. The letter outlined the agreed-upon expectations and shared goals such as referral targets, scholarly opportunities (i.e., white papers, informative handouts, and publications), and educational programs that could be customized to meet the specific needs of each community. The document provided BRANCH and extension agents a clear guide to co-construct a mutually beneficial partnership.

All FACE personnel worked on building community relationships prior to data collection to work toward achieving short-term (e.g., recruitment) and long-term goals (e.g., community engagement). Initial in-person meetings with extension agents and community liaisons were held to gain a deeper understanding of the community. The BRANCH team set a goal to consistently spend time in the community to build relationships with community members and gain a deeper understanding of local strengths.

##### 2.2.2.2 Building relationships

The FACE team prioritized building meaningful, trust-based relationships with families and the broader community. Team members spent dedicated time volunteering in local settings to deepen community connections and demonstrate long-term investment. Once participants were enrolled, the study’s Certified Child Life Specialist took an individualized, family-centered approach to participation, using their expertise to create a supportive and positive experience tailored to each family’s needs. These strategies aimed not only to foster trust, but also to support snowball recruitment, a process in which current participants refer others from their social networks to join the study (31). Importantly, the team also worked to establish reciprocal relationships, ensuring that community voices and needs were integrated into the research process.

Through discussions with community liaisons, the research team was able to identify the values and needs of the community and offer time and resources to support initiatives that are central to the community. Examples of these reciprocal connections included hosting child-friendly educational activities and programs (see Appendix 1 for example material) for libraries, homeschool groups, and community resource fairs. The BRANCH research team also gave community presentations to students, physicians, and community groups on topics such as parenting, behavior, neuroscience, and community resilience. Additionally, students from local schools were invited to tour the research lab to learn about neuroscience.

In April 2024, the team received feedback from the community that Spanish research material was needed. In response, one Spanish-speaking team member, one Spanish-speaking research assistant, and one Spanish-speaking community liaison collaborated to translate material and contribute to the team’s efforts in building cultural competence. The research team worked with the Spanish-speaking community liaison to generate materials that address cultural perceptions and hesitations about research.

##### 2.2.2.3 Maintaining rapport

The current study is set to span across 6 years of data collection (2023–2029). To maintain rapport in an effort to support retention, the team has taken a multifaceted approach that includes (1) continuous participant communication between data collection periods, (2) regular contact with extension agents, whose deep community connections and local knowledge help guide the research team in understanding community needs, and (3) sustained connection with community liaisons and organizations.

To strengthen long-term presence and communication with participants between data collection visits the team maintains an active social media presence. The researchers share a variety of content, including practical parenting tips, family resources, and videos about the study with the goal of providing value for families. A newsletter with study updates, community events, and family activities, designed to engage families, is also distributed. Personal birthday and holiday cards are also sent out to reinforce that families are valued as part of a community, not just as study subjects.

Lastly, the research team prioritizes long-term involvement with community organizations. This includes consistently showing up to community events, keeping in contact with the leaders of community organizations, and holding regular meetings with community liaisons to adjust the engagement protocols. Additionally, the team is able to tailor their activities and offerings to the community based on a better knowledge of community needs, strengths and interests.

### 2.3 Measures of community, family and participant engagement

Although evaluating engagement protocols was not an original objective of the parent study, the research team maintained detailed records that were utilized for the current analysis. Additionally, supplementary data and feedback were collected to further support this investigation, as described below.

#### 2.3.1 Ongoing records

The research team kept detailed records of the following: number of participants enrolled each month, demographics of participants (age, gender, race, and ethnicity), the number of phone calls and emails made to interested families prior to enrollment, the methods through which enrolled participants were recruited into the study, and the number of community events attended. Recruitment methods included (1) referrals from participants, staff, community liaisons, or extension agents, (2) sign-ups at community events, (3) flyers, (4) social media, (5) clipboarding, (6) the study website, (7) past participants from other lab studies, or (8) connections through other research groups. The research team also kept lists of participants potentially interested in participating, meaning they had signed up for more information at a tabling event, clipboarding, or through the study’s website, but had not yet enrolled.

#### 2.3.2 Trust survey

Following completion of wave one of the BRANCH study, participants were sent an anonymous survey via Qualtrics (32) to their email asking about their trust in research. The trust survey consisted of three close-ended questions, and three open-ended questions. The first three questions were, “How much did you trust research studies *before* participating in the BRANCH research study?,” “How much did you trust research studies *after* participating in the BRANCH research study?,” and “At this point in time, how much do you trust the research team? The research team members are the people you interacted with over the phone, at your home, or at the imaging center on campus.” These questions had a response scale from 1 (*not a lot*) to 5 (*completely*). The open-ended questions were: “Could you please share an experience that stands out about your family’s participation in the study?,” “What part(s) of the experience would you change for next time?” and “What could researchers do to help build trust in this study?”

#### 2.3.3 Qualitative reflection notes

Research team members kept non-structured, detailed notes and reflections after each community event, participant visit and discussions with community liaisons. These notes documented staff observations and feedback on participant engagement, recommendations from community liaisons, challenges encountered, and overall event or data collection visit dynamics. Additionally, they captured contextual factors such as environmental influences, participant comfort levels, and any adaptations made to enhance engagement or address emerging challenges.

#### 2.3.4 Feedback from extension agents

A university community health engagement coordinator on the research team conducted semi-structured interviews with five extension agents to gather feedback on BRANCH’s community engagement process. During these interviews, the extension agents shared their experiences and provided insights on collaborating with the BRANCH team. The interview guide included the following questions: (1) “Please describe what you did with or for the BRANCH study,” (2) “What went well about your experience with the BRANCH study?,” (3) “What did not go well, or was challenging about working with the BRANCH study?,” (4) “What would you change in the future to make things work better?,” (5) “Establishing trust with communities is important for researchers. What about your experience with the BRANCH study encouraged building trust with the community? What did not help build trust with the community?,” and (6) “How would you feel about sharing information about other research opportunities with members of your community?” The agent interviews were conducted virtually, recorded, and transcribed using Zoom^2^ video conferencing software.

### 2.4 Qualitative analyses and descriptive statistics

All descriptive analyses and visualizations were conducted using R (33). Descriptive statistics were used to examine the number of participants enrolled each month, the demographic breakdown of enrolled participants, and the recruitment methods used. The qualitative data collected from the trust survey, reflection notes, and extension agent feedback were analyzed using MAXQDA (34), which facilitated the systematic coding and categorization of responses. We employed both an inductive and deductive thematic analysis approach (35), guided by the continuum model of community engagement (28), to identify and organize key themes related to trust in the research process, shared decision-making, capacity building, sustainability of partnerships, and bidirectional communication. Additional themes related to factors influencing community partner and member participation that emerged from the data were also noted.

A team-based approach was used to ensure consistency and validity in the coding process, with three team members (a FACE team member, the project coordinator, and an extension agent) independently reviewing and discussing the data to reconcile any discrepancies. The interpretation of the results involved were collaborative where the team discussed the meaning and implications of the themes, drawing on both the data and insights from their involvement with the community to contextualize the findings.

## 3 Results

### 3.1 Findings on community engagement

As of November 2024, the FACE team established connections, meaning they had communicated with, attended or plan to attend events, or hosted community outreach activities with 110 community organizations and contacts, including Departments of Public Health, nonprofit family-centered organizations, community members, and community leaders who continue to support recruitment and retention efforts. Two main themes emerged from extension agents’ feedback on efforts to partner with the community. The first theme was about the BRANCH study building trust through consistent presence. Extension agents expressed that the time BRANCH staff spent in the community, alongside extension agents and community leaders, conveyed respect for the dedication necessary to cultivate local connections and earn the trust of community members. One extension agent noted,

“I don't know if this is a testament to the study or the testament to the people. But when they [FACE Team] said they wanted to show up. They showed up. And I think that's really huge. Even when we had one event at the school, and it was kind of like last minute, but they were there. And I think showing up consistently in a community that doesn't know you is important. And they did that…which I think is important in the gaining of trust.”

The second theme to emerge was role ambiguity. Some extension agents expressed challenges in understanding their specific role within the study. Additionally, some agents felt their expertise and status as faculty members were not fully acknowledged or utilized by the research team.

Feedback from community liaisons on study community engagement protocols resulted in three primary recommendations. The first was regarding the language used in study materials (e.g., changing “rural” to “small town” and “home visit” to “family visit”), and the approach to the study, shifting from a deficit approach to a resilience approach [e.g., asking, “what makes small towns so strong/resilient?” (36)]. The second recommendation was about the resources to offer back to the community. Community liaisons recommended a range of resources such as parenting classes, internship opportunities for high schoolers, school-visits to teach science lessons and hosting community activities at fundraisers or local non-profit events. After offering these resources back to the community, the team transitioned from *asking* to attend community events to *being asked* to attend community events. The third recommendation was regarding barriers to participation. Community liaisons suggested that the team should offer transportation and child-care, as these may be two primary barriers for rural families interested in participating.

### 3.2 Findings from participant recruitment and enrollment

From November 2023 to November 2024, the research team collected the contact information from 530 individuals who had expressed interest in participating. During this time, the team made/sent a total of 1,407 phone calls, emails and/or texts to 467 interested participants. On average, it took 2.22 contact attempts (e.g., phone calls, emails, texts) to successfully enroll a participant in the study.

**Figure 4:**
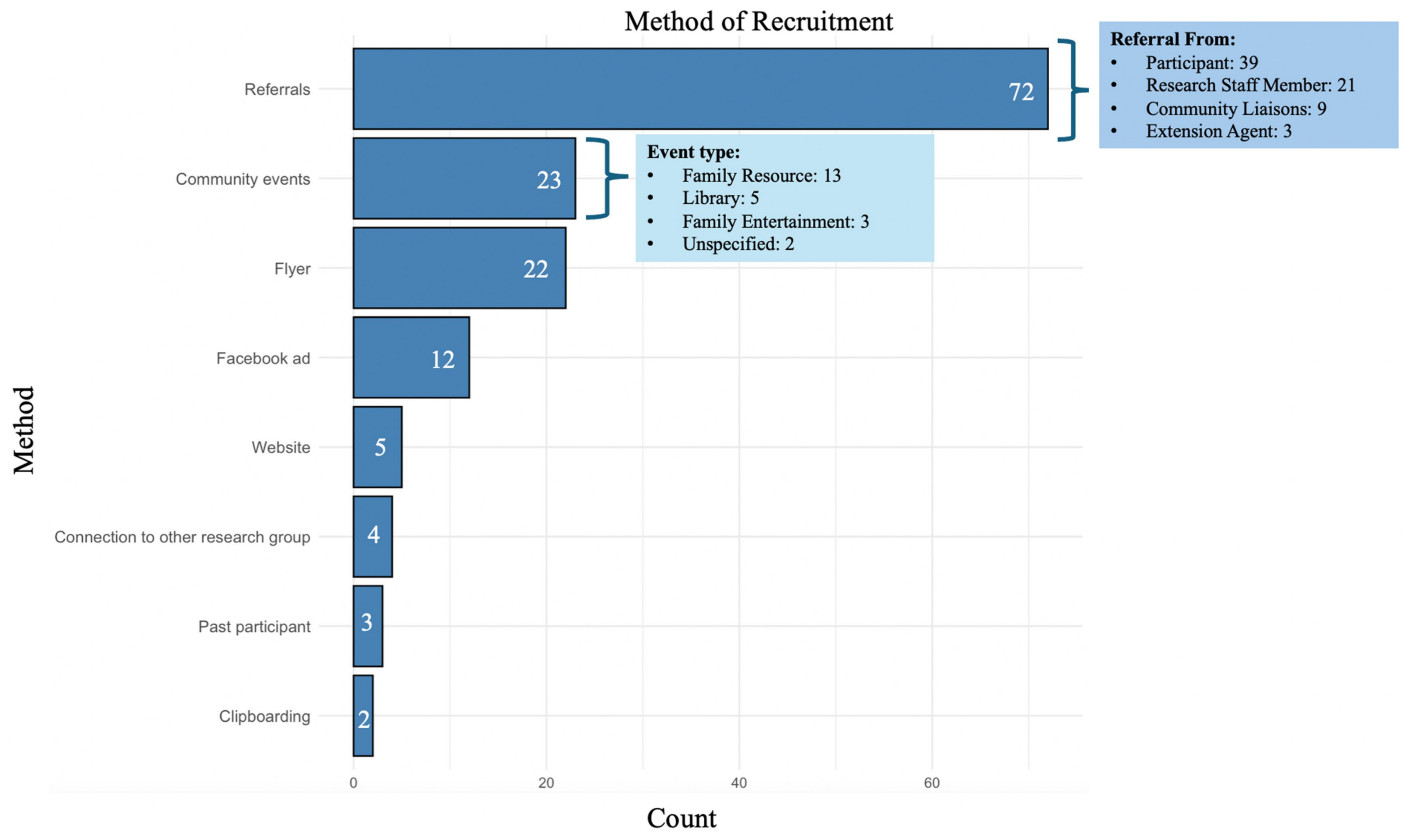
The method of recruitment reported by study participants.

Since recruitment began in November 2023, 146 participants have been enrolled in the study following IRB approval. Of those initially interested in participating, 135 were ineligible, 153 did not respond to contact attempts, 40 were no longer interested, and 56 were still being contacted or in the enrollment process at the time of manuscript writing (November, 2024). Of the participants enrolled who specified the method in which they were recruited into the study (*N* = 145; see Figure 4), the most successful method was referrals (49.66%), followed by community events (15.86%), and flyers posted with community partners (15.17%). The least effective recruitment methods included connections with other research groups, past participation, and clipboarding.

**Figure 5:**
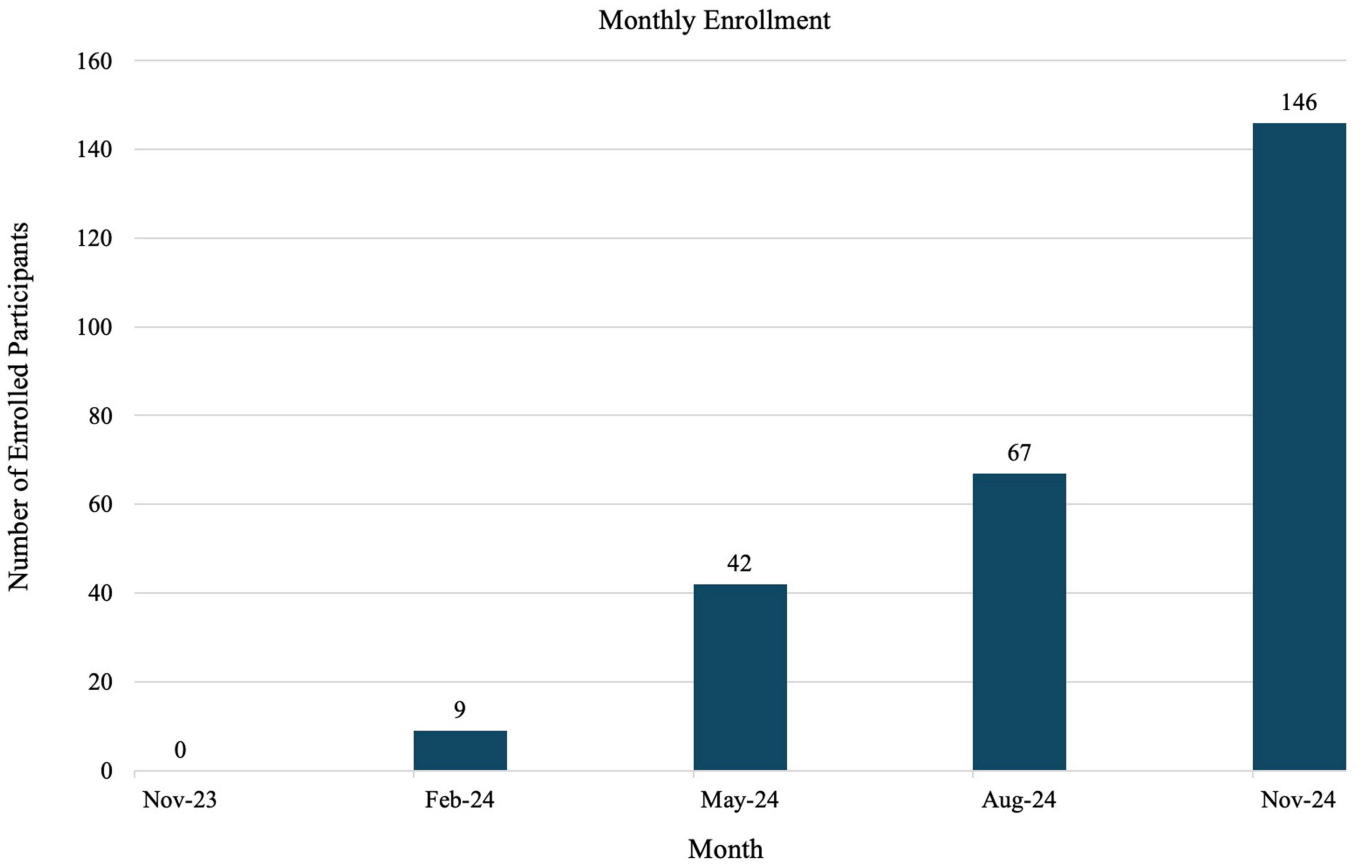
Enrollment numbers from November 2023 to November 2024.

Figure 5 depicts the cumulative enrollment numbers from November 2023 to November 2024. Participation increased by 117% in three months from August to November.

To provide a broader context for the sample, we averaged the racial, ethnic, and sex demographic information across the 12 counties from which our participants were recruited. Table 1 shows the demographic makeup of (1) the community from which BRANCH sampled from, (2) the BRANCH sample, and (3) the respondents to the trust survey. Table 1 shows racial representation in BRANCH generally reflective of the broader community among White, Black, and Native Hawaiian or Pacific Islander individuals. The BRANCH study had fewer participants who identified as Asian, American Indian or Alaskan Native, Other or Hispanic/Latino(a) compared to the larger community. Additionally, the trust survey respondents were over representative of White participants compared to the larger community.

**Table 1:** Demographic characteristics of participants.

Demographic	Average community demographics (*N* = 1,454,952)	Branch study sample child (*N* = 146)	Trust survey respondents caregiver (*N* = 26)
Race/Ethnicity N (%)			
White	736,393 (50.61%)	81 (56%)	20 (77%)
Black or African American	403,866 (27.76%)	41 (28%)	3 (11%)
Asian	144,343 (9.92%)	6 (4%)	1 (4%)
Native Hawaiian or Pacific Islander	920 (0.06%)	1 (1%)	1 (4%)
American Indian or Alaskan Native	10,106 (0.69%)	0 (0%)	0 (0%)
Prefer not to say	NA	1 (1%)	0 (0%)
Other	159,324 (10.95%)	2 (1%)	0 (0%)
Hispanic/Latino(a)	275,005 (18.90%)	13 (9%)	1 (4%)
Sex N (%)			
Female	745,476 (51.24%)	58 (47%)	NA
Male	709,476 (48.76%)	65 (53%)	NA
Age Min-Max M (SD)	NA	6–10 years 8.5 years (0.8 years)	NA

M, Mean; SD, Standard Deviation; Min, Minimum; Max, Maximum; NA, Not available.

Reflection notes provided by the Latina community liaison highlight skepticism among Latina/o participants. Many expressed hesitations about the university’s affiliation and concerns over how their data would be used. One liaison recalled a participant apologizing for not confirming a visit, explaining, *“…[I] got scared and a bit worried about sharing her “[her child’s]” information…”* Another participant expressed, *“no confiamos en nadie”* (“we do not trust anyone”). The liaison felt they eased concerns by discussing their own Mexican heritage, emphasizing the importance of authentic cultural connections in building trust. The participants referenced above both completed wave one of data collection.

### 3.3 Findings from the trust survey

**Figure 6:**
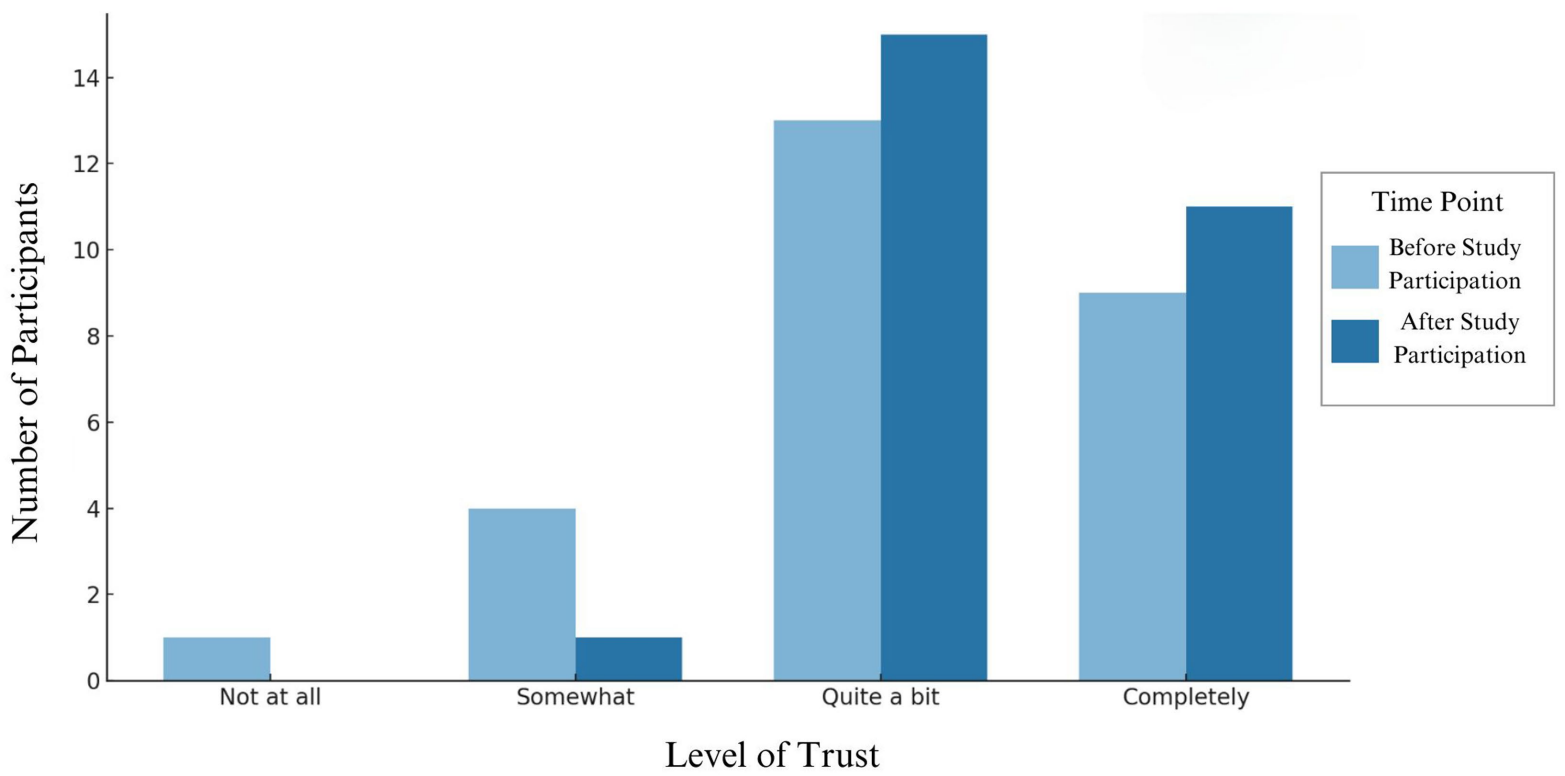
Participant level of trust in research before and after current study participation.

Results on pre and post study trust from the 27 participants who completed the trust survey can be seen in Figure 6. The demographic makeup of this sample can be seen in Table 1. No participants reported a decrease in trust. Ten participants’ response remained at “quite a bit,” nine remained at “completely,” one changed from “not at all” to “quite a bit,” three changed from “somewhat” to “quite a bit,” and four changed from “quite a bit” to “completely.”

The first theme to emerge in qualitative data from the trust survey was the increased comfortability stemming from the family-centered approach. When responding to the question, “Could you please share an experience that stands out about your family’s participation in the study?,” 85% of respondents specifically referenced the family-centered approach set in place by the Certified Child Life Specialist and how it made them/their child feel comfortable in their experience. One responded, *“I was so impressed by how comfortable they made my daughter feel. Seeing her name on the board when she walked in, the instructions they shared and the stuffed animal they gave her lessened her worries (and mine!)”* and another noted *“I appreciate the care the BRANCH team took in explaining things to my son and making sure he felt comfortable and consented to all of the activities he participated in.”*

The second theme to emerge was staff warmth and professionalism in interacting with children. One caregiver said, “*The researchers were so gentle with my daughter and spoke and acted in ways that indicated that they were trustworthy to her and me and helped put us at ease through the whole process.”*

When asked what the team can change for future studies, respondents noted (1) making the study shorter, (2) improving physical comfortability with some study material (i.e., more comfortable chairs, and earphones), and (3) ensuring participants are made aware of any findings from the study. When asked for recommendations on building trust in this study, respondents recommended explaining how all the components of the study will be connected in the research (i.e., the “why”) and discussing with them how this research will ultimately benefit their community.

## 4 Discussion and recommendations for future research

Many developmental science studies employ complex, longitudinal designs that demand significant efforts in recruitment and retention. The success of these studies hinges on sustained engagement and strong partnerships with the communities involved. The research team implemented a multifaceted, iterative approach to build trust and foster meaningful engagement through reciprocal interactive processes with communities. The current study aimed to describe and reflect on the community engagement, recruitment and retention strategies utilized over the course of 1 year in a complex longitudinal study on child and family health.

The research team spent the first year of the project connecting with over one hundred community organizations. The capacity to discuss the project with community liaisons, learn about the communities’ values and needs, and engage with various organizations was reliant on having a team with protected time dedicated to this work. Coming into initial meetings prepared to listen to community leaders and offering meaningful resources can positively influence how the research team is received (12, 37).

Cultivating a positive relationship with extension agents as a means to connect authentically to the community was a major goal of the research team. Extension agent feedback suggests that the consistency and time commitment BRANCH staff demonstrated by attending events was beneficial to this working relationship. However, some extension agents expressed challenges in understanding their specific roles within the study and felt their expertise as faculty members was not fully acknowledged or utilized. This lack of clarity may have limited their ability to effectively connect FACE staff to the community and to educate the team about local context and needs. By more fully recognizing and leveraging the scientific and community-based expertise of extension agents, research teams can better align study goals with community priorities (38). Future studies may benefit from clearer role definitions and greater collaboration with extension agents by involving them more actively in the research design and implementation processes (38).

Additionally, studies should consider forming dedicated engagement teams with full-time staff, ideally from the community being studied, to sustain meaningful connections (28, 39). Although not a resource available to the research team, health extension regional offices and agents [see (40) for an example] aim to connect resources to rural and underserved communities and can provide valuable community health services similar to agricultural extension agents. Future studies should consider leveraging these resources to enhance community engagement and health outreach in rural settings.

In addition to community engagement efforts, participant recruitment and enrollment requires significant time and resources. The research team made over one thousand calls and emails, with an average of 2.22 contact attempts per successful enrollment. Referrals emerged as the primary method of participant enrollment. Future studies should consider offering referral incentives to encourage participation, particularly in rural or close-knit communities where word-of-mouth connections are particularly influential (41).

A snowball effect was observed, with the sample more than doubling in three months, approximately 10 months after the recruitment launch. Despite successful recruitment in the first year of the study, for every eligible participant successfully recruited, there were more than two who declined to participate. Recognizing the time and effort required to build community trust and generate referrals is essential for successful community engagement (39, 41). Funding agencies should consider these timelines when planning grant opportunities to ensure adequate support for long-term engagement.

Once enrolled, qualitative feedback suggests that the family-centered approach facilitated by the Certified Child Life Specialist and the staff’s warmth and professionalism were key factors in fostering participant trust. While most participants’ trust levels remained stable, 33% reported increased trust in research after participating. Although the exact reasons for increased trust are unclear, prior research indicates that familiarity and positive experiences can enhance trust (42, 43). Incorporating family and child-centered, participant-focused approaches may be beneficial in future studies to further strengthen participant relationships.

The BRANCH study had lower participation rates among Latino/a individuals compared to the broader community. Qualitative feedback suggests mistrust may have contributed to this underrepresentation, consistent with the literature (11). Community liaisons highlighted that having researchers with shared cultural heritage and providing clear, accessible explanations about data use and privacy measures could help build trust and promote engagement in research. Future recruitment efforts should prioritize cultural humility, deeper understanding of diverse communities, and ongoing learning to promote inclusive participation. This includes building relationships with community leaders, adapting recruitment strategies to be culturally responsive, and addressing barriers to participation, such as financial constraints and mistrust (11).

Lastly, participant feedback from the trust survey indicates the importance of reducing participant burden and increasing the study’s scientific communication efforts. Existing studies have found that removing logistical barriers can facilitate recruitment and retention (41, 44). Previous research, as well as insights from the current study’s community liaisons, suggest that transportation and childcare challenges can significantly hinder participation in rural areas (41, 44). The BRANCH study addresses these barriers by offering gas compensation and childcare for siblings during study visits. Other strategies to reduce burden may include avoiding reliance on government-issued identification, incorporating Spanish-speaking staff, providing meals or snacks, and offering flexible scheduling options (44). Qualitative feedback from participants also emphasizes the importance of receiving updates on study findings. Future studies should prioritize transparent, accessible dissemination strategies to ensure communities understand how research outcomes may benefit them [see (45)].

### 4.1 Limitations

While the current study provides valuable insights, several limitations must be acknowledged. First, recruitment and retention are not direct proxies for trust, which is a critical factor influencing research participation. Future studies should explicitly examine trust within populations involved in research to better understand its role and impact.

Second, there is no retention data, preventing the evaluation of the effectiveness of these strategies for maintaining participant engagement throughout the study. This gap limits the ability to provide concrete recommendations for improving retention in similar research contexts. Additionally, eligibility criteria were expanded during the study, which undoubtedly influenced recruitment patterns. Despite this, the team believes that documenting the protocol used can still provide valuable guidance for other researchers conducting longitudinal and complex studies. Finally, the sample was predominantly White. Previous research has demonstrated that racial and ethnic minorities often harbor deeper mistrust of research due to historical injustices and systemic inequities (11). Addressing this mistrust is essential for fostering greater inclusivity and diversity in future studies.

### 4.2 Conclusion

Trust-building in science should go beyond transactional goals, fostering connections that empower communities and create shared ownership of the research process. Although building trust is necessary for recruitment and maintaining trust is essential for retention, trust also plays a crucial role in ensuring the validity and reliability of the data collected. Establishing genuine, reciprocal relationships with community members and leaders, such as extension agents, school board members, and other stakeholders, can enhance participant engagement and data integrity. The research team attempted to ground trust in mutual respect, transparency, and a commitment to continuously increase community involvement while amplifying the strengths and voices of community members. By incorporating feedback and prioritizing the community’s needs, the research team aims to ensure that the study is both inclusive and equitable.

While the current efforts have demonstrated promising results, it is important to acknowledge that this process is iterative and requires a reciprocal exchange of information with the participant community. The research team is still learning and refining approaches, actively seeking input from colleagues and the community to enhance research methods. Although the foundation of community involvement provided a strong starting point, more work remains to fully achieve the study’s goals of employing community-based participatory research (CBPR) to build trust and foster deeper community engagement (28). The research team is committed to progressing along a community engagement continuum, striving to embrace the principles of authentic community-based participatory research. By doing so, the team aims to deepen collaboration and co-ownership with community partners, advancing research that is not only rigorous but also deeply rooted in the priorities and experiences of those it seeks to serve. Future research should prioritize these trust-building practices to enhance recruitment, retention, and ethical representation of diverse populations in developmental science.

## Data Availability

The datasets presented in this article are not readily available because data requests can be sent to the principal investigator for review. Requests to access the datasets should be directed to oshri@uga.edu.

## Footnotes

^1^ https://childlifeoncall.com

^2^ https://www.zoom.com/home/
