# Correction: Building trust in rural communities: recruitment and retention strategies in developmental science

**DOI:** 10.3389/fpubh.2025.1640525

**Published:** 2025-06-24

**Authors:** Ava Reck, Lauren Holley, Kyle Bower, Sarah Whitaker, Caroline Hall, Courtney Brown, Alison Berg, Caroline Alvarado, Diane Bales, Katharine Suma, Kimberly Fowler, Charles Geier, Assaf Oshri

**Affiliations:** ^1^Department of Psychology, University of Oregon, Eugene, OR, United States; ^2^Department of Human Development and Family Science, University of Georgia, Athens, GA, United States; ^3^Extension and Outreach, University of Georgia, Athens, GA, United States; ^4^Department of Nutritional Science, University of Georgia, Athens, GA, United States; ^5^Department of Psychology, New York University, New York, NY, United States; ^6^Office of Research, University of Georgia, Athens, GA, United States

**Keywords:** building trust, rural communities, recruitment strategies, retention strategies, developmental science, community engagement, rural engagement, recruitment and retention

In the published article, there was an error in the Funding statement. An applicable funding source (the National Center for Advancing Translational Sciences of the National Institutes of Health under Award Number UL1TR002378) was mistakenly omitted by the authors. The original text said: “The author(s) declare that financial support was received for the research and/or publication of this article. This work was supported by the National Institute of Drug Abuse under award number R01 DA055630-03. The content is solely the authors' responsibility and does not necessarily represent the official views of the National Institutes of Health.”

The correct Funding statement appears below.

