# Supplementary material for: Building trust in rural communities: recruitment and retention strategies in developmental science
Source: Front Public Health. 2025 May 7;13:1586988. doi: 10.3389/fpubh.2025.1586988 (PMC12092468; doi:10.3389/fpubh.2025.1586988)
Supplement: Supplementary file 1 [file Table_1.DOCX]

**Appendix One**

Appendix 1 from the manuscript, “Building Trust in Rural Communities: Recruitment and Retention Strategies in Developmental Science” submitted to *Frontiers in Public Health*, Special Issue: The Erosion of Trust in the 21st Century: Origins, Implications, and Solutions

The following appendix lists the tools used by the Family and Community Engagement team, and the Child Life Specialist to improve participant engagement and comfortability in the study. Links to more information is proved when available.

Appendix 1.

| **Tool and Reference Link** | **Project Use** |
| --- | --- |
| SupportSpot App  Link: [Child Life on Call, LLC](https://childlifeoncall.com/supportspot/)  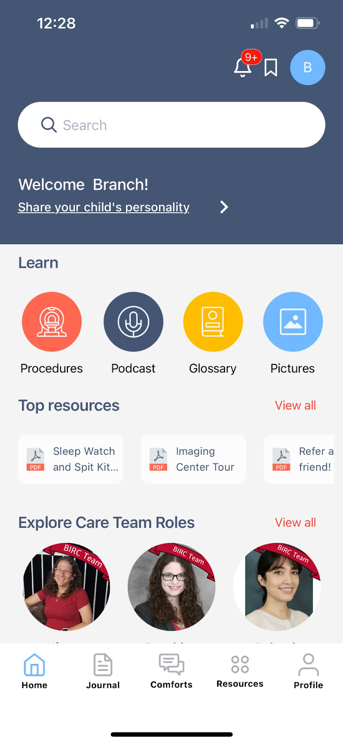 | ***SupportSpot App.*** Offered to participants to download on their personal device at no cost. Provided information on the research team, links to resources and study instructions, Q&A’s regarding MRI’s and study participation, and reminders about study sessions. |
| MRI Sticker Board  Link: [Hope for Henry](https://hopeforhenry.org/)  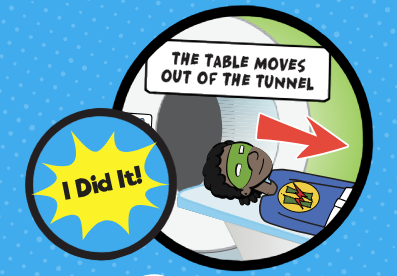 | ***MRI Sticker Board.*** A game board that uses child-friendly images and terminology to explain the MRI. The board also allows children to place stickers as they progress through the study. |
| Virtual MRI Preparation Session  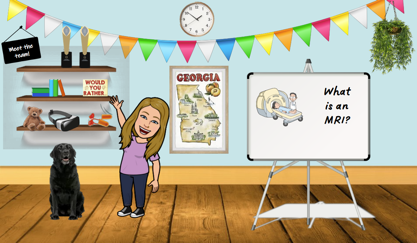 | ***Virtual MRI Preparation Session.*** This virtual preparation center was hosted online |
| Engaging Participant Materials  Link: [Center Website](https://www.gadevelopmentalscience.com/resources)  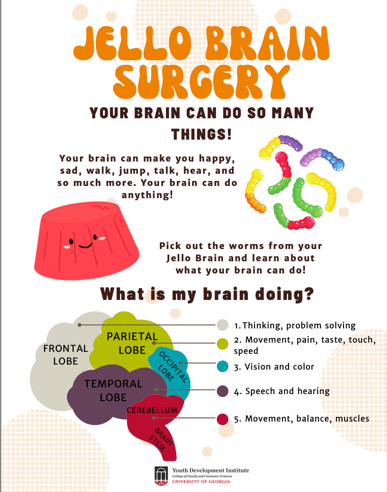 | ***Engaging Participant Materials.*** Examples of activities the research team offered at community events including homeschool groups, S.T.E.M events, and family resource events. |
| Virtual Reality MRI Education and Simulator  Link: [KindVR](https://www.kindvr.com/)  Link: [GetWell Pals](https://www.getwellpals.co/)  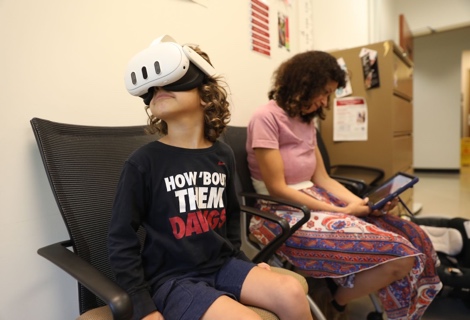 | ***Virtual Reality MRI Education and Simulator.*** This stimulator was used both at community events, to introduce children to S.T.E.M, but also as a preparatory tool. The headset stimulated an MRI, including MRI sounds and feedback on staying still. |
| Individualized Welcome Sign  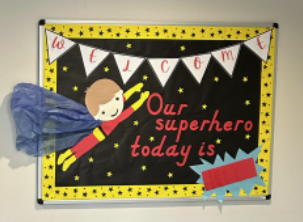 | ***Individualized Welcome Sign.*** This sign was developed to be placed outside of the MRI imaging center. The participant’s name is placed on the billboard to create a child-friendly and welcoming environment at the start of the MRI visit. |
